# DICAM in the Extracellular Vesicles from Astrocytes Attenuates Microglia Activation and Neuroinflammation

**DOI:** 10.3390/cells11192977

**Published:** 2022-09-24

**Authors:** Jin Han, Hyun-Jung Cho, Donghwi Park, Seungwoo Han

**Affiliations:** 1 Laboratory for Arthritis and Cartilage Biology, Research Institute of Aging and Metabolism, Kyungpook National University, Daegu 41404, Korea; 2 Laboratory for Arthritis and Bone Biology, Fatima Research Institute, Daegu Fatima Hospital, Daegu 41404, Korea; 3 Department of Physical Medicine and Rehabilitation, Ulsan University Hospital, University of Ulsan College of Medicine, Ulsan 44521, Korea; 4 Division of Rheumatology, Department of Internal Medicine, School of Medicine, Kyungpook National University, Daegu 41404, Korea

**Keywords:** DICAM, neuroinflammation, astrocyte, microglia, CRPS, extracellular vesicle

## Abstract

Cross-talk between astrocytes and microglia plays an important role in neuroinflammation and central sensitization, but the manner in which glial cells interact remains less well-understood. Herein, we investigated the role of dual immunoglobulin domain-containing cell adhesion molecules (DICAM) in the glial cell interaction during neuroinflammation. DICAM knockout (KO) mice revealed enhanced nociceptive behaviors and glial cell activation of the tibia fracture with a cast immobilization model of complex regional pain syndrome (CRPS). DICAM was selectively secreted in reactive astrocytes, mainly via extracellular vesicles (EVs), and contributed to the regulation of neuroinflammation through the M2 polarization of microglia, which is dependent on the suppression of p38 MAPK signaling. In conclusion, DICAM secreted from reactive astrocytes through EVs was involved in the suppression of microglia activation and subsequent attenuation of neuroinflammation during central sensitization.

## 1. Background

Central sensitization is a condition of the nerve system, which is in a state of persistent high reactivity, caused by a pathologic process that occurs at the level of the dorsal horn of the spinal cord, and is critical for the development and persistence of chronic pain, such as complex regional pain syndrome (CRPS), postherpetic neuralgia, and fibromyalgia [1,2,3]. Accumulating evidence suggest that central sensitization is driven by neuroinflammation that is characterized by the infiltration of leukocytes, activation of glial cells, and increase in inflammatory mediators [4,5,6]. In the process of neuroinflammation, microglia play a critical role as the tissue-resident macrophages of the central nervous system (CNS) [4]. In physiologic conditions, microglia regulate CNS development and homeostasis via neuronal–microglial interactions, apoptotic cellular debris clearing, trophic factor secretion, and synaptic modeling [7]. However, prolonged stimulation of microglia leads to the release inflammatory cytokines, such as TNF-α, IL-6, IL-1β and prostaglandin E2, reactive oxygen species (ROS), and excitotoxins, including glutamate [8]. These neuromodulators ultimately enhance pain signal transmission by facilitating glutamate-mediated nociceptive neurotransmission and contributing to the loss of gamma-aminobutyric acid and glycin-secreting inhibitory interneurons [9,10,11].

Astrocytes, as the most abundant glial cell type in CNS, have diverse functions, such as structurally forming the blood–brain barrier and supporting neuronal cells, and functionally recycling neurotransmitters, supplying nutrients to the neuronal cells, and controlling the ion concentration of the CNS microenvironment [12]. Moreover, recent evidence has reported the significant contribution of astrocytes in neuroinflammation as crucial regulators of innate and adaptive immune responses in the injured CNS [12,13]. The reactive astrocytes have the phenotypes of hypertrophic processes and upregulation of glial fibrillary acidic protein (GFAP), and secretion of inflammatory cytokines, chemokines, and neurotransmitters [14]. In addition, reactive astrocytes were found to release extracellular vesicles (EVs) enriched with active biomolecules, such as endosomal proteins, miRNA, and DNA, which can be involved in intercellular communication [15]. Although various cells in the CNS can excrete EVs, recent data have shown that astrocytes act as the major source of EVs in vivo and in vitro [16,17]. In addition, the inhibition of EV-release in astrocytes by knockdown of Rab27a increases the number of active microglia, suggesting an immunomodulatory function of astrocyte-released EVs [16]. Furthermore, EVs from astrocytes can be taken up by microglia, and induce an impairment of phagocytosis [18] and promote M2 differentiation of microglia [19]. However, other studies have reported microglia activation with astrocyte-derived EVs via TLR7-NF-kB signaling, suggesting a context-dependent pro- or anti-inflammatory role of astrocyte-derived EVs [20].

Dual immunoglobulin domain-containing cell adhesion molecule (DICAM), also known as limitrin and matrix remodeling associated 8 (MXRA8), is a type I transmembrane protein with two V-type Ig domains that was originally found in the end-feet of astrocytes that form the blood–brain barrier [21,22]. It has phylogenetic homology with the junctional adhesion molecule family and is ubiquitously expressed in various cell types, including macrophages, Th17 cells, endothelial cells, epithelial cells, and chondrocytes [22,23,24,25,26,27]. Our group has found that DICAM as a ligand for αVβ3 integrin contributes to angiogenesis, osteoclast differentiation and barrier function of mucosal epithelial cells. In addition, DICAM functions as a receptor for arthritogenic alphaviruses in multiple joint cell types, such as synovial fibroblasts, osteoblasts, chondrocytes, and skeletal muscle cells [28]. A recent study revealed that DICAM is mainly expressed on circulating Th17 cells in patients with active multiple sclerosis (MS), and promotes Th17 cell trafficking across the blood–brain barrier through homophilic and heterophilic interactions with DICAM and αVβ3 integrin, respectively [24]. However, the role of DICAM in central sensitization can be different from that of MS as an autoimmune-mediated demyelinating disease, where T- or B-cells are critical.

The aim of the present study was to investigate whether DICAM involves the pain behaviour and glial activation during central sensitization of the CRPS model in vivo. We also compared the phenotypes of neuroinflammation and related signaling pathways in mixed glial cells from DICAM knockout (KO) mice and wildtype (WT) littermates in vitro. Finally, we focused on the cellular source of DICAM in mixed glial cells and confirmed EV-mediated delivery mechanisms.

## 2. Materials and Methods

### 2.1. Animal and Mouse Models of CRPS

C57BL/6J male mice at 12 weeks of age were purchased from a commercial supplier of the Koatech Experimental Animal Center (Pyeongtaek, Korea), and DICAM knockout (KO) mice were provided by Professor T. Yonezawa (Okayama University, Okayama, Japan). The mouse model of CRPS was induced in 12-week-old male mice by using the tibial fracture and cast immobilization method. Briefly, under general anesthesia with 1.5% isoflurane, the left distal tibia of DICAM KO mice and their wildtype (WT) littermates were fractured using a hemostat and immediately wrapped with casting tapes. Tramadol (30 mg/kg) and enrofloxacin (5 mg/kg) were injected subcutaneously for the next 2 days, and casts were maintained for 3 weeks, before removing them under general anesthesia. Pain-related behaviors were then assessed once a week for over 7 weeks postoperatively, and the mice were euthanized for the histologic analysis of the L3-L5 dorsal horn of the lumbar spinal cord [6]. All animal experiments were approved by the Institutional Animal Care Committee of Kyungpook National University (Approval No. KNU-2019-63/24), and were conducted in accordance with the animal care guidelines of the National Institutes of Health.

### 2.2. Hind Paw Volume Measurement

The hind paw volume was measured every week after cast-off as follows: paw volume (mm^3^) = 1/2(length of long axis × length of short axis × thickness of paw) [6]. The lengths of the long axis, short axis, and thickness of the ipsilateral and contralateral hind paws were measured using an electronic digital caliper [6].

### 2.3. Threshold Punctate Mechanical Stimulation (von Frey Test)

The von Frey test was conducted to evaluate tactile allodynia using calibrated monofilaments (von Frey hairs; Stoelting, Wood Dale, IL), which were applied to the plantar surface of the ipsilateral and contralateral hind paws placed on an elevated maze in the acrylic cage. Paw withdrawal was considered as a positive response, and the 50% withdrawal threshold upon six repeated applications of varying force with the von Frey filament was measured using the up-down method [29].

### 2.4. Spontaneous Weight-Bearing Test (Incapacitance Test)

Spontaneous weight bearing on the hind limbs, also known as the incapacitance test, was conducted to assess the downward force applied by each hind limb using the incapacitance meter (SangChung commercial, Seoul, Korea). Briefly, mice were placed in the restrainer, and the hind limbs were rested on the two weight averaging platform pads. The paw pressure of each hind limb underwent 10-s measurements approximately 10 times, and the results were averaged. Data were expressed as the percentage of weight distributed on the ipsilateral hind limb.

### 2.5. Rotarod Test

The rotarod test was performed to measure the locomotor performance of mice using the LE8205 Accelerating Rota Rod (Havard Apparatus, Holliston, MA, USA). Briefly, mice were placed on a rotating cylinder with an increasing speed of 0–30 rotations per minute (rpm) for 60 s, which was followed by an additional 240 s at 30 rpm [30]. Latency to fall measurements were repeated five times with 30 min breaks, and the results were averaged [30].

### 2.6. Immunofluorescence Staining

Mice were perfused with phosphate buffered saline (PBS) through the aorta to remove blood, and the lumbar spinal cord was subsequently dissected. After fixation with 4% paraformaldehyde, the spinal cord specimens were cryoprotected in 30% sucrose for 2 days, embedded in the optimal cutting temperature compound, sliced into 20 μm-thick sections and mounted on gelatin-coated slides. Tissue sections were washed with tris-buffered saline with Tween-20 (TBST), blocked with 10% donkey normal serum (cat. #ab7475, Abcam, Cambridge, UK) in 0.3% Triton X-100 for 60 min, and then incubated with primary antibodies against Iba-1 (rabbit, 1:200; Wako, Osaka, Japan) and glial fibrillary acidic protein (GFAP) (rabbit, 1:500; cat. Z0334, DAKO, Carpinteria, CA, USA) overnight at 4 °C. Subsequently, the sections were incubated with fluorescein isothiocyanate (FITC-) or Cy5-conjugated secondary antibodies (1:500, Jackson ImmunoResearch, West Grove, PA, USA) for 2 h, and slides were washed and mounted with Fuoromount aqueous mounting medium (cat.#F4680, Sigma) [5]. Four square areas of the ipsilateral and contralateral dorsal horn of the spinal cord were randomly selected and photographed at 100 magnifications under a KI-3000F fluorescence microscope (Korealabtech, Gyeonggi-do, Korea). The Iba-1 and GFAP-positive cells were quantified using ImageJ software (NIH; https://imagej.nih.gov/ij/ accessed on 2 February 2020). In order to distinguish the background and positive cells, the binary threshold was set to 50%, and positive cells were defined as more than 5 pixels.

### 2.7. Primary Cell Isolation and BV2 Microglia Cell Line Culture

Primary mixed glial cells were prepared from the brain cortices of 2- to 3-day-old newborn pups among the C57BL/6J mice. After removing the meninges, brain tissues were homogenized and mechanically disrupted with the nylon mash, which were triturated to the cell suspension and centrifuged at 200 g for 15 min. After resuspension, cells were plated onto T-75 flasks in the DMEM with 10% FBS medium, cultured for 14 days and used for the in vitro experiment. Primary mixed glial cells were then stimulated with lipopolysaccharides (LPS) (100 ng/mL) and interferon-gamma (IFN-γ) (10 ng/mL) in 96-well plates for 24 h.

For the isolation of primary astrocytes, mixed glial cells that were cultured for 14 days were agitated by shaking them at 250 rpm overnight to remove weakly attached microglia. Culture media containing cells were discarded, and astrocytes were dissociated using trypsin-EDTA and then collected by centrifuging them at 1500 rpm for 5 min. For the isolation of primary microglia, mixed glial cells that were cultured for 14 days were separated from the glia layer by shaking them at 200 rpm for 3 hours. Afterwards, the floating microglia were collected by centrifuging them at 1500 rpm for 5 min. The purities of primary astrocytes and microglia exceeded 95%, as determined by GFAP and CD11b, using traditional reverse transcription polymerase chain reaction (RT-PCR).

Murine microglial cell line BV2 was obtained from Xiehe Medical University (Beijing, China). The cells were cultured in DMEM supplemented with 10% heat-inactivated fetal bovine serum, 100 IU/mL penicillin, 100 mg/mL streptomycin and 2 mmol/L glutamine.

### 2.8. Nitric Oxide (NO) Quantification

Nitric oxide colorimetric assay was performed as described previously. The quantity of nitrite, a stable metabolite of NO, in the culture medium was measured as an indicator of NO production. Briefly, 100 μL of cultured media was mixed with the same amount of Griess reagent (Cell Signaling Technology, Danvers, MA, USA) in 96-well plates, incubated at room temperature for 10 min, and the absorbance at 540 nm was measured in a microplate reader (BioTek, Winooski, VT, USA). Fresh culture medium was used as a blank in every experiment, and the quantity of nitrite was determined from a sodium nitrite standard curve.

### 2.9. Real-Time Quantitative PCR and Reverse Transcription PCR

Total RNA was isolated from cultured primary mixed glia, astrocytes, microglia, and the BV-2 microglial cell-line using the TRIzol reagent (Invitrogen). Briefly, cDNA was synthesized from 2 μg of total RNA using the Superscript II reverse transcription kit (Invitrogen, Waltham, MA, USA), with a random hexameter and oligo d(T) primer. Real-time qPCR was then performed using the Luna^®^ Universal One-Step RT-qPCR Kit (New England Biolabs, Ipswich, MA, USA), which was followed by detection using the ViiA™ 7 Real-Time PCR System (Applied Biosystems, Foster City, CA, USA). Additionally, the 2-ΔΔCT method was used to calculate the relative changes in gene expression, with glyceraldehyde 3-phosphate dehydrogenase (GAPDH) as the control. All the RT-qPCR reactions were performed in triplicate and repeated two to three times. Among them, the representative results are shown. The primer nucleotide sequences are listed in Table 1.

Reverse transcription PCR amplification using specific primer sets was carried out at an annealing temperature of 55–60 °C for over 20–30 cycles using the Applied Biosystems Veriti™ Thermal Cycler (Applied Biosystems). To analyze the PCR products, 10 μL of each PCR was electrophoresed on 1% agarose gel stained with ethidium bromide and was detected using the Criterion Stain-Free Imager (Bio-Rad).

### 2.10. Enzyme-Linked Immunosorbent Assay (ELISA)

For cytokine measurements, primary mixed glial cells were stimulated, and the supernatant was subjected to ELISA analysis for mouse TNFα (Abcam; #ab208348, Cambridge, MA, USA), IL-6 (Abcam; #ab46100, Cambridge, MA, USA) and IL-10 (Abcam; #ab100697, Cambridge, MA, USA). The absorbance read was performed using a microplate reader at 450 nm, and the results were compared against a standard curve. Furthermore, the DICAM levels of cultured supernatant and plasma were measured using the DICAM ELISA kit (MyBioSource; #MBS907149, San Diego, CA, USA) according to the manufacturer’s instructions.

### 2.11. Western Blot Analysis

Total cell lysates or nuclear and cytoplasmic extracts were separated by sodium dodecyl-sulfate polyacrylamide gel electrophoresis (SDS-PAGE) and transferred onto polyvinylidene difluoride (PVDF) membranes (Amersham Biosciences, Buckinghamshire, UK). The membranes were blocked with 5% bovine serum albumin at room temperature for 1 h and were incubated with the aforementioned primary antibodies for another hour with shaking. Primary antibodies against p38 MAPK, p-p38 MAPK, JNK, p-JNK, ERK1/2, p-ERK1/2, Akt, p-Akt, IκBα, p-IκBα, p65, p-p65, Stat3, and p-Stat3 were purchased from Cell Signaling Technology (Beverly, MA, USA); antibodies against CD14, CD68, and CD63 were purchased from Cusabio Technology (Houston, TA, USA); the ERK antibody was purchased from BD Biosciences (Franklin lakes, NJ, USA); and the β-actin antibody was purchased from Sigma Aldrich (St. Louis, MO, USA). After the membranes were washed, incubation with the horseradish peroxidase (HRP)-conjugated secondary antibody was carried out for 1 h. The membrane signal was then visualized using Supersignal Chemiluminescent Substrates (Thermo Fisher Scientific, Waltham, MA, USA), and protein-specific signals were detected using the LAS-3000 (Fujifilm, Tokyo, Japan). Western blot band intensities were quantified with ImageJ software.

### 2.12. Blood Sampling from CRPS Patients and Controls

A pilot study to compare the serum DICAM level in CRPS patients with controls was conducted on 5 CRPS patients who visited the Rehabilitation clinic of Daegu Fatima hospital between May 2019 and June 2020 and fulfilled the Budapest diagnostic criteria for CRPS. The 5 controls were matched to cases on age and sex. The average disease duration of CRPS patients was 20.6 ± 9.7 months and numeric rating scale of pain intensity was 7.4 ± 1.1 (Table 2). The plasma samples drawn from CRPS patients and controls were stored in a refrigerator at −80 degree Celsius, and were subjected to ELISA analysis within 1 month of blood collection. The study was conducted in accordance with the Declaration of Helsinki, and approved by the Institutional Review Board of Daegu Fatima Hospital (DFH18ORIO358, 2018-10-10).

### 2.13. Isolation of Extracellular Vesicles

To obtain astrocyte-derived exosomes, the primary astrocytes were cultured at a density of 2 × 10^7^ cells in a T175 flask and stimulated with or without LPS (100 ng/mL) and IFN-γ (10 ng/mL) by incubation for 24 h at 37 °C. After reaching 80% confluency, astrocytes were rinsed with PBS and cultured with serum-free medium for 72 h. The obtained supernatants were then filtered through a 0.22-μm filter and centrifuged at 10,000× *g* for 30 min at 4 °C to remove cellular debris. Subsequently, supernatants were collected and ultracentrifuged at 100,000× *g* for 1 h. The remaining supernatants were subsequently discarded, and the pellets were washed and resuspended in 200 μL of PBS.

### 2.14. Astrocyte-Derived Conditioned Media Treatment

To obtain astrocyte-derived conditioned media, primary astrocytes were plated at a density of 2 × 10^6^ cells/well in 6-well plates and stimulated with or without LPS (100 ng/mL) and IFN-γ (10 ng/mL) for 24 h. The cells were then washed with PBS and cultured in fresh DMEM for an additional 24 h. Afterwards, conditioned media were collected and centrifuged at 320× *g* for 2 min to remove cellular debris, and the resulting astrocyte-conditioned media were treated with mixed glia cells for 24 h.

### 2.15. Statistical Analysis

All data are presented herein as mean ± standard error of the mean (SEM). Statistical analysis to compare the mean values of two groups was performed using the Mann–Whitney U test, a nonparametric test, as the sample size was small and it could be assumed as normal distribution. *p*-values ≤ 0.05 were considered statistically significant. Statistical analyses were performed with Prism software, version 8.0 (GraphPad Software, La Jolla, CA, USA).

## 3. Results

### 3.1. DICAM KO Mice Showed Enhanced Nociceptive Behavior and Increased Number of Astrocytes and Microglia in the Dorsal Horn of the CRPS Model

**Figure 1:**
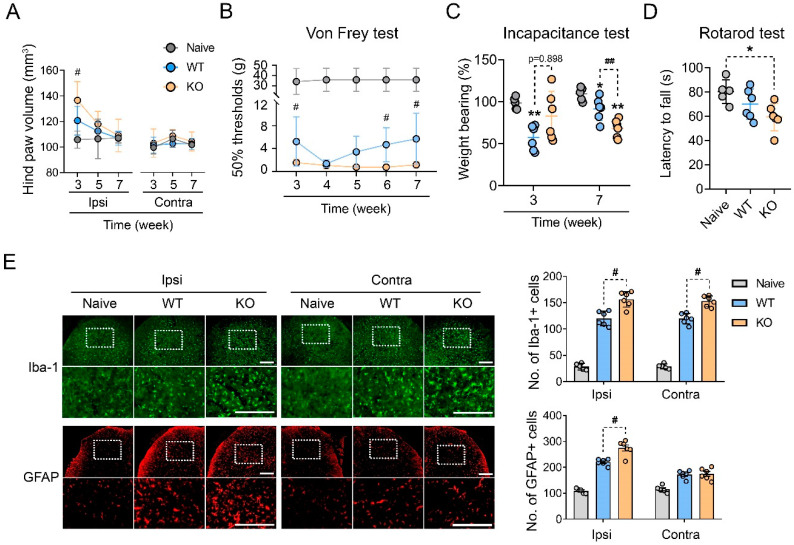
Loss-of-function of DICAM leads to severe pain-associated behavioral phenotypes in the tibia fracture and cast immobilization-induced CRPS model. (**A**) The volume of the ipsilateral (Ipsi) and contralateral (Contra) hind paws of DICAM KO mice and their WT littermates was measured at 3, 5, and 7 weeks postoperatively. *n* = 6 per group. (**B**) Mechanical allodynia was assessed using the von Frey test from the 3rd to 7th week in DICAM KO mice and their WT littermates. *n* = 6 per group. (**C**) The weight bearing on hind paw was assessed using the incapacitance test at the 3rd and 7th weeks. The weight bearing percent represents the ratio of the weight bearing of the ipsilateral and contralateral hind paws. Thus, any percentage <100% represented hind limb unweighting. *n =* 6 per group. (**D**) The rotarod was used to measure the locomotor capacity of CRPS mice at the 7th week. *n* = 3 per group. (**A**–**D**) * *p* < 0.05, ** *p* < 0.01 as compared with naïve mice, and # *p* < 0.05, ## *p* < 0.01 as compared with WT littermates by Mann–Whitney U test. (**E**) Representative figures of immunofluorescence staining for microglia (Iba-1; a microglial marker) and astrocytes (GFAP; an astrocyte marker) in the dorsal horn of the lumbar spinal cord of CRPS mice. The dashed area of upper panel was enlarged and displayed in the low panel. Scale bar indicates 1 μm. Iba-1 and GFAP fluorescence-positive cells were quantified and expressed as the ratio to DAPI-positive cells. # *p* < 0.05 by Mann–Whitney U test. *n* = 4 per group.

To elucidate whether DICAM is involved in the central sensitization associated with neuropathic pain, the tibia fracture-cast immobilization (fracture/cast) model of CRPS was induced in 12-week-old DICAM KO mice and their WT littermates. The ipsilateral hind paw volume reached its maximum at three weeks after the fracture/cast procedure and then reduced and the DICAM KO mice showed a significant increase in ipsilateral hind paw volume at 3 weeks compared to WT (Figure 1A). The tactile allodynia of the hind paws assessed by von Frey filaments revealed that the von Frey withdrawal threshold was lower in DICAM KO mice than WT mice at 3 weeks, which became similar in 4th and 5th week, and then worsened again in the DICAM KO mice in the 6th and 7th week (Figure 1B). The weight-bearing of the ipsilateral hind limb was not different in WT and DICAM KO mice at the acute phase (3rd week after fracture), but it significantly decreased in DICAM KO mice compared to WT at the chronic phase of CRPS at 7 weeks (Figure 1C). The rotarod test examining motor coordination behaviour revealed that DICAM KO mice showed a shorter latency to fall compared to naïve mice, suggesting an impaired motor coordination, while WT mice could hang on to the rotarod for a similar time compared to the naïve mice (Figure 1D).

As this enhanced pain-associated behavior suggests a sensitization of nociceptive pathways, we checked the activation of glial cells in the spinal cord dorsal horn (SCDH). The immunostaining of a microglia marker, Iba-1, revealed that DICAM KO mice had a significant increase in Iba-1 positive cells in both the ipsilateral and contralateral side of SCDH compared to WT mice (Figure 1E). The GFAP immunostaining for astrocytes that are the most abundant glial cells in the CNS also showed an increase in GFAP-positive cells in ipsilateral SCDH of DICAM KO mice, but not in the contralateral side (Figure 1E). These results demonstrate that DICAM is involved in glial cell activation during central sensitization associated with CRPS.

### 3.2. DICAM Deficiency Aggravates Proinflammatory Glial Cell Responses to Inflammatory Stimuli

**Figure 2:**
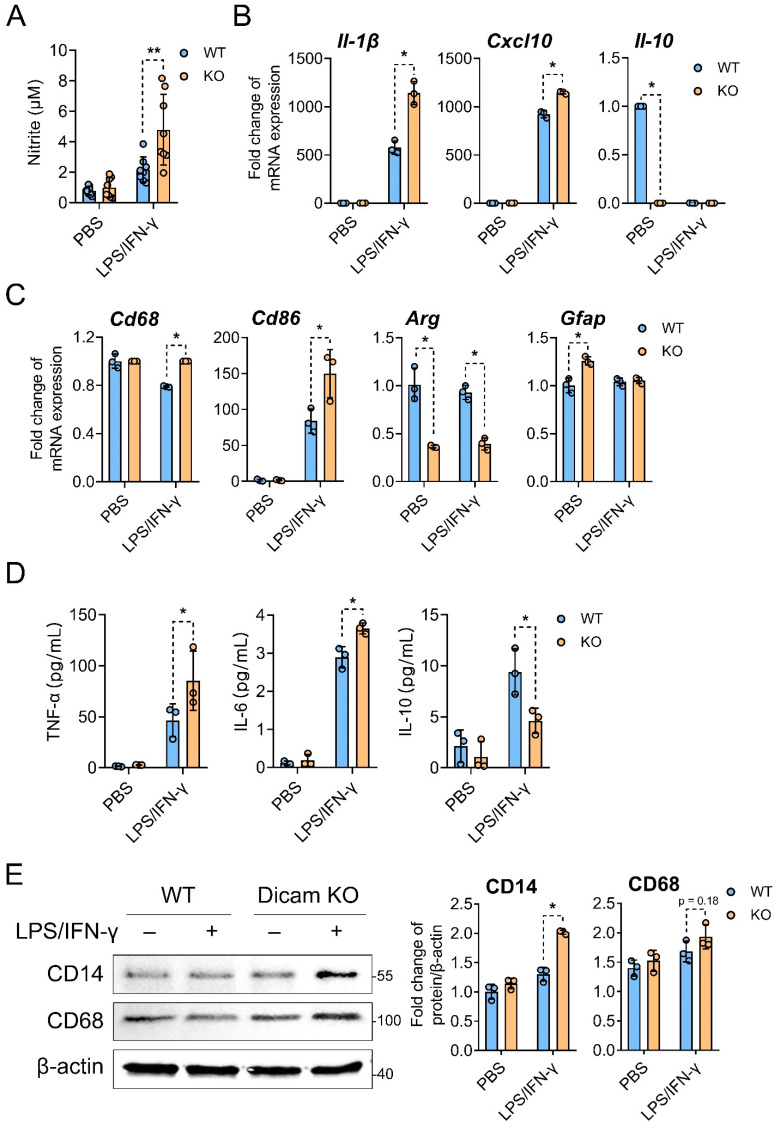
Deficiency of DICAM promotes the neuroinflammatory phenotype in primary mixed glia culture. (**A**,**D**,**E**) Primary mixed glial cells composed of astrocytes (~80% GFAP+ cells) and microglia (~20 % OX-42+ cells) were isolated from DICAM KO mice and their WT littermates. These cells were treated with LPS (100 ng/mL) and IFN-γ (10 ng/mL) for 24 h, and the PBS-treated group was used as a negative control. (**A**) The nitrite concentration in the culture supernatant was analyzed with the Griess reagent reaction and estimated from a standard absorbance at 540 nm. *n* = 8 per group. (**B**,**C**) Primary mixed glial cells from DICAM KO mice and their WT littermates were treated with LPS and IFN-γ for 6 h. (**B**) Expressions of proinflammatory (IL-1β, CXCL10) and anti-inflammatory cytokines (IL-10), (**C**) M1-like marker CD86, M2-like marker Arg1, astrocyte marker GFAP, and microglia marker CD68 were analyzed using real-time RT-qPCR. All the RT-qPCR reactions were performed in triplicate and repeated for two to three times. Among them, the representative results are shown. (**D**) Pro- (TNF-α, IL-6) and anti-inflammatory cytokines (IL-10) were quantified using ELISA in the cell culture supernatants. *n* = 3 per group. (**E**) CD14 and CD68 protein levels were determined by Western blot analysis and were quantified using the ImageJ program. (**A**–**E**) * *p* < 0.05 and ** *p* < 0.01 by Mann–Whitney U test. *n* = 3 per group.

Given the increase in microglia and astrocytes in DICAM KO mice in SCDH of the CRPS model, the inflammatory glial cell phenotype was evaluated under the pro-inflammatory stimuli by LPS and IFN-γ in vitro. Nitrite, a stable metabolite of nitric oxide that is a major mediator of innate immune responses, was significantly increased in the mixed glial cells from DICAM KO mice in the presence of LPS and IFN-γ, but not in unstimulated glial cells (Figure 2A). This pattern was reproduced in the RNA expression of proinflammatory cytokines, such as IL-1β and CXCL10. The expression of IL-10 was reduced to almost no expression in mixed glial cells from DICAM KO mice under unstimulated conditions, and in both DICAM KO and WT glial cells under the LPS and IFN-γ stimulated condition (Figure 2B). The RNA of CD68, a marker for activated microglia, and CD86, a marker for M1 microglia, were upregulated in DICAM KO mixed glial cells compared to WT in the stimulated condition, while that of Arg-1, a marker for M2 macroglia, decreased in DICAM KO glial cells in both stimulated and unstimulated conditions. GFAP, a marker for astrocytes, increased in DICAM KO mixed glial cells compared to WT in unstimulated conditions, but not in LPS and IFN-γ stimulated conditions (Figure 2C). When the pro- and anti-inflammatory cytokines were assessed using ELISA analysis, IL-6 increased in the supernatants from DICAM KO mixed glial cells, compared to WT in stimulated conditions. TNF-α level showed an increasing trend in DICAM KO glial cells in stimulated conditions, but failed to show statistical significance. The anti-inflammatory cytokine, IL-10, was decreased in DICAM KO compared to WT mixed glial cells in stimulated conditions (Figure 2D). The protein level of CD14, a marker for primed microglia, was upregulated in DICAM KO glial cells in the stimulated condition but not in the unstimulated condition, while CD68, another marker for activated microglia, failed to show any difference between DICAM KO and WT mixed glial cells (Figure 2E). These results collectively suggest that DICAM is involved in the anti-inflammatory phenotypes of mixed glial cells and in the M2 polarization of microglia, especially under inflammatory milieu.

### 3.3. DICAM Is Involved in the Activation of p38 MAPK in Mixed Glial Cells but Not in Astrocytes

**Figure 3:**
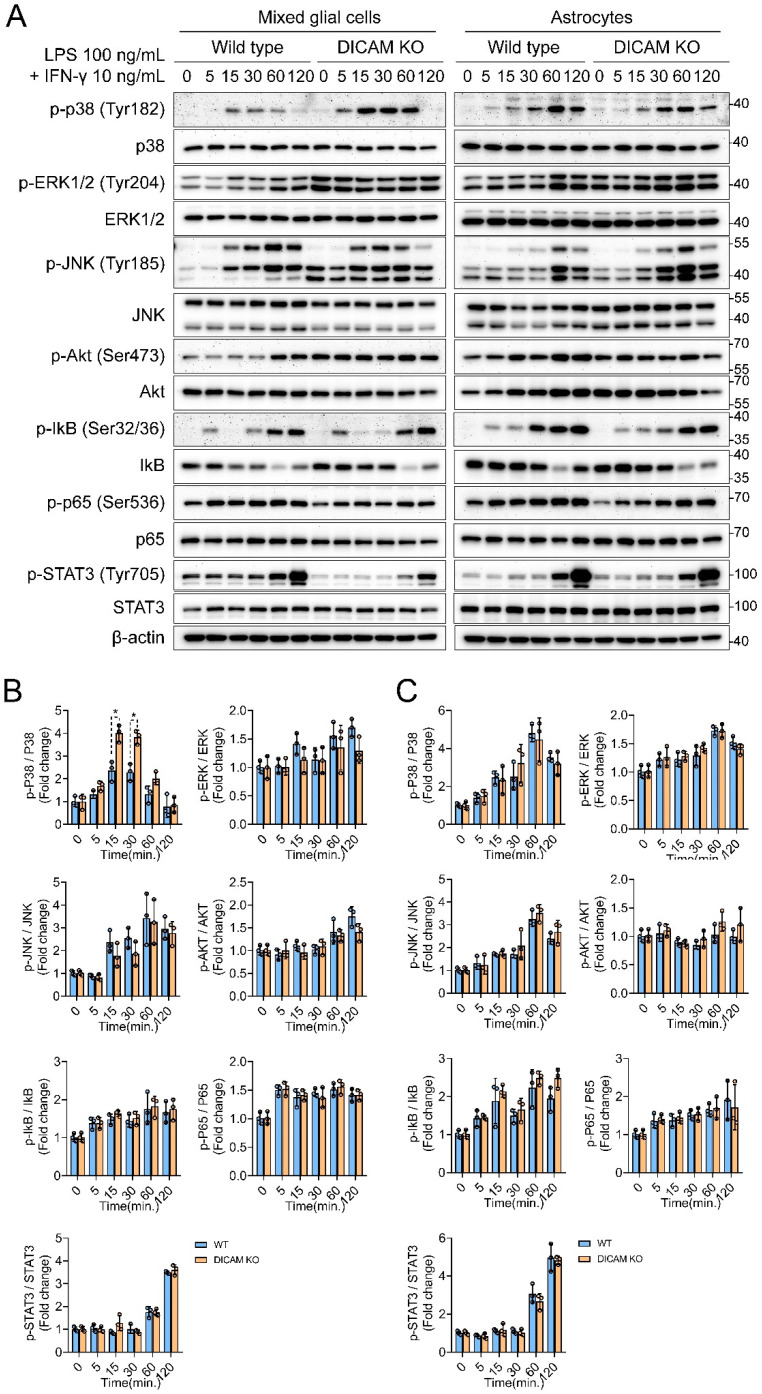
Deficiency of DICAM promotes p38 MAPK activation in primary mixed glial cell culture but not in astrocytes. (**A**) Starved primary mixed glial cells and astrocytes derived from DICAM KO mice and their WT littermates were stimulated with 100 ng/mL of LPS and 10 ng/mL of IFN-γ for an indicated time. Phosphorylation of p38, ERK1/2, JNK MAP Ks, AKT, IkB, p65, STAT1, and STAT3 were determined using Western blot analysis. Experiments were performed in triplicate and representative blot is displayed. (**B**,**C**) The Western blot band intensities of phosphorylated proteins were quantified by densitometric analysis on the Image J program and normalized to non-phosphorylayed total protein, which was displayed as relative densitometric bar and dot graphs. * *p* < 0.05 by Mann–Whitney U test. *n* = 3 per group.

To elucidate the signaling mechanisms that are involved in the DICAM-mediated anti-inflammatory phenotypes of mixed glial cells, we analyzed the major signaling pathways activated by LPS and IFN-γ in DICAM KO and WT mixed glial cells and astrocytes. Mixed glial cells derived from DICAM KO mice revealed an enhanced phosphorylation of p38 MAPK compared to WT by LPS and IFN-γ, but did not show significant difference in other types of signaling, including ERK1/2, JNK, AKT, NF-kB, and STAT3 (Figure 3). In contrast, astrocytes from DICAM KO and WT mice did not show any significant difference in the signaling cascade by LPS and IFN-γ (Figure 3). Considering that mixed glial cells are mainly composed of astrocytes and microglia, these results suggest that the altered p38 MAPK activation of microglia was responsible for the proinflammatory phenotypes in DICAM KO mixed glial cells.

### 3.4. Activated Astrocytes Secrete DICAM through Extracellular Vesicles

**Figure 4:**
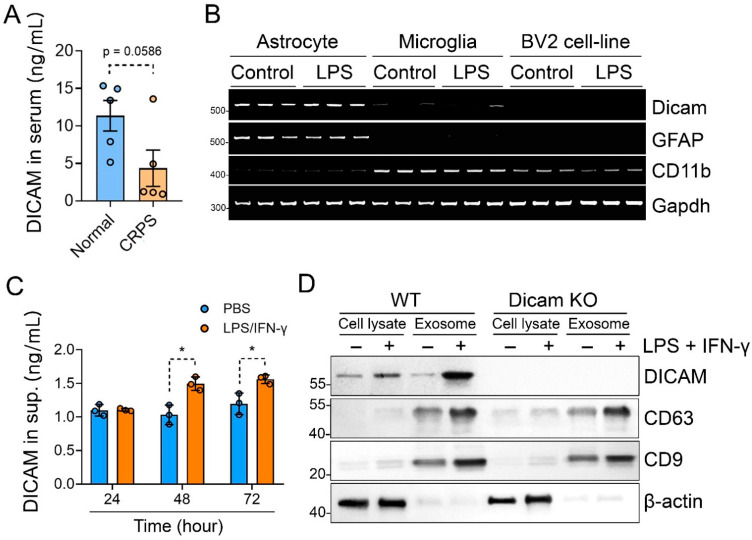
DICAM is highly enriched in the exosome from stimulated primary astrocytes. (**A**) Serum DICAM levels from patients with CRPS and age- and sex-matched healthy controls were determined using ELISA analysis and compared by Mann–Whitney U test. *n* = 5 per group. (**B**) RT-PCR analysis for DICAM, GFAP (a marker for astrocyte), and CD11b (a marker for microglia) were performed with mRNA isolated from primary astrocytes, primary microglia, and murine microglia cell-line BV-2. (**C**) The protein level of DICAM in the culture supernatants of WT primary astrocytes was measured using ELISA analysis. Primary astrocytes were treated with 100 ng/mL of LPS and 10 ng/mL of IFN-γ for 24 h. * *p* < 0.05 by Mann–Whitney U test. *n* = 3 per group. (**D**) Representative Western blot image of the total cell lysates and exosomes secreted by primary astrocytes in the medium showing DICAM and CD63 expressions (a marker for exosome).

We then checked the serum level of DICAM with ELISA assay in CRPS patients to assess the role of DICAM as a serum biomarker compared to healthy participants. It showed a tendency to decrease in CRPS patients compared to the healthy controls (*p* = 0.059) (Figure 4A). To further examine the production of DICAM in glial cells, we assessed the RNA expression in primary astrocytes, primary microglia, and murine microglia cell-line BV2. When compared to GFAP, an astrocyte marker, and CD11b, a microglia marker, DICAM was selectively expressed in astrocytes but not in microglia (Figure 4B). These findings indicate that DICAM mainly produced by astrocytes affects the activation of microglia in Figure 3 and also suggest that DICAM acts in a paracrine manner. To prove this hypothesis, we checked the DICAM protein level in the supernatant of the primary astrocyte culture. Stimulation with LPS and IFN-γ significantly increased the level of DICAM in the culture supernatant of primary astrocytes at 48 and 72h (Figure 4C). Subsequently, we assessed whether this increase in DICAM in the culture supernatant of primary astrocytes was through extracellular vesicles (EVs). Immunoblot for CD63 and CD9, a marker for EVs, revealed that the activated astrocytes by LPS and IFN-γ secreted more exosomes than unstimulated astrocytes. Interestingly, DICAM was significantly enriched in the EVs isolated from the supernatant of activated astrocytes compared to unstimulated exosomes or total cell lysate (Figure 4D). Taken together, these results suggest that activated astrocytes secret DICAM-enriched EVs, which are involved in the regulation of microglia activation.

### 3.5. DICAM from Activated Astrocytes Acts as Coupling Factor That Regulates Mixed Glial Cell Activation

**Figure 5:**
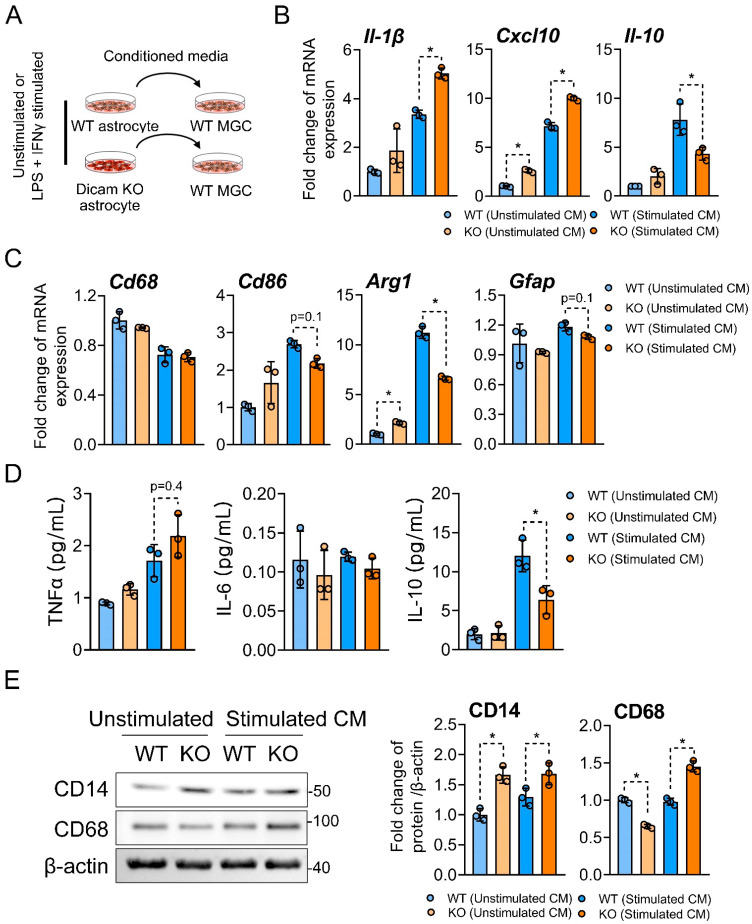
Conditioned media from stimulated DICAM KO astrocytes recapitulates an inflammatory phenotype in primary mixed glial cell culture. (**A**) Primary astrocytes from DICAM KO mice and their WT littermates were stimulated with 100 ng/mL of LPS and 10 ng/mL of IFN-γ for 24 h and then replaced with serum-free media. After 24 h, the conditioned media were collected and treated to the primary mixed glial cell culture from WT mice. (**B**,**C**) Quantitative PCR analysis of mRNA for IL-1β, CXCL10, IL-10, CD86, Arg1, Gfap, and CD68 in the primary mixed glial cell culture treated with the conditioned media from WT or DICAM KO mixed glial cells for 6 h. (**D**) ELISA analysis of TNFα, IL-6, and IL-10 secreted by primary mixed glial cells that were treated with the conditioned media for 24 h. *n* = 3 per group. (**E**) Immunoblot analysis of total protein extract from mixed glial cells treated with the conditioned media for 24 h. The bar graph depicts the means ± SD of the band intensity of CD14 and CD68, normalized to β-actin from three independent experiments. *n* = 3 per group. (**B**–**E**) * *p* < 0.05 by Mann–Whitney U test.

We further examined the influences of the conditioned media from DICAM KO and WT astrocytes on the inflammatory phenotypes of the mixed glial cell culture. Conditioned media from DICAM KO astrocytes activated by LPS and IFN-γ significantly increased mRNA expression of *IL-1β* and *CXCL10* from mixed glial cells, while it decreased that of *IL-10* (Figure 5A,B). It also decreased the expression of *Arg-1*, a marker for M2-microglia, but failed to affect *CD68*, *CD86* and *GFAP* expression (Figure 5C). In the ELISA analysis of pro- and anti-inflammatory cytokines, conditioned media from stimulated DICAM KO astrocytes significantly reduced secreted IL-10 levels in glial cells, but did not affect TNFα and IL-6 (Figure 5D). When we assessed microglia activation with the CD14 and CD68 immunoblot, the stimulated conditioned media from DICAM KO astrocytes increased both CD14 and CD68 expressions in mixed glial cells, but the unstimulated media showed opposite effects on CD14 and CD68 (Figure 5E).

## 4. Discussion

**Figure 6:**
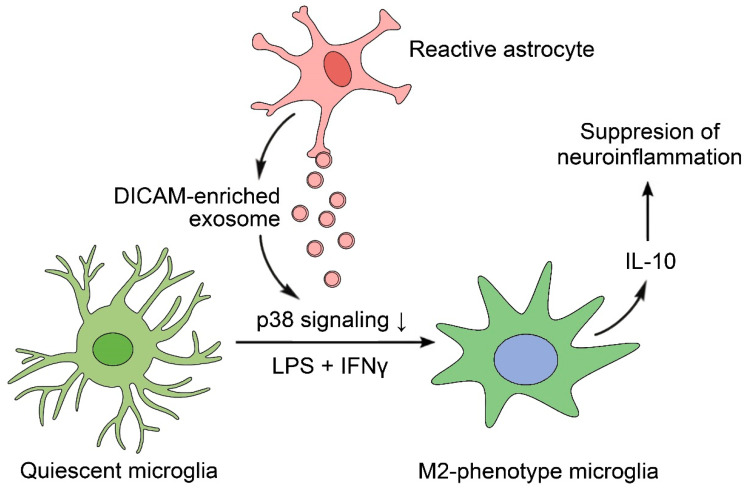
The scheme derived from the present study. Reactive astrocytes secrete DICAM-enriched exosomes in an inflammatory milieu, which polarizes quiescent microglia to anti-inflammatory M2 microglia in the presence of LPS and IFNγ.

A growing body of evidence has shown the cross-talk between astrocytes and microglia in neuroinflammation, but the manner in which glial cells interact remains unclear. Our findings demonstrated the critical role of the astrocyte-specific protein, DICAM, as a paracrine modulator of microglia activation. DICAM is selectively secreted in reactive astrocytes via EVs and contributes to the regulation of neuroinflammation through the M2 polarization of microglia, which is dependent on the suppression of p38 MAPK signaling. Indeed, DICAM KO mice show enhanced nociceptive behavior and glial cell activation in the spinal cord of the CRPS model, suggesting its significant role in the central sensitization of pain (Figure 6).

Cross-talk between astrocytes and microglia critically contributes to CNS homeostasis in response to damage or degeneration of neurons [31]. Although DICAM is predominantly expressed in astrocytes, immunologic stimuli with LPS and IFN-γ did not show any differences in the signaling cascade of DICAM KO and WT astrocytes. Instead, our data showed an activation of p38 MAPK by LPS and IFN-γ in DICAM KO mixed glial cells, which consisted of 20% of microglia, and 80% astrocytes [32]. Together with the increase in M1 microglia markers observed in our DICAM KO glial culture, this result suggests the altered activation of microglia in DICAM KO mice and a role of DICAM as a cross-talk molecule that regulates the activation of microglia. An increasing amount of evidence revealed that cross-talk between astrocytes and microglia is mainly mediated by secretory mediators, such as cytokines, growth factors and neurotransmitters [31]. Moreover, EVs from reactive astrocytes play crucial roles in this reciprocal cross-talk, which can display a combination of neurotoxic or neuro-protective properties in a context-dependent manner [33]. However, very little is known about the molecules that regulate the EV-mediated cross-talk, which can act as a double-edged sword. Our results suggest that DICAM-enriched EVs from astrocytes can play a neuro-protective role by regulating the activation of microglia.

From the viewpoint of interaction with microglia, we need to investigate the ways in which DICAM-enriched EVs from astrocytes work. Given that DICAM is mainly located at the membrane fraction of cells [25], it is more likely to be present in the membrane of EVs rather than in the cytoplasm. Our group reported that DICAM is involved in cell adhesion through homophilic interaction with DICAM itself and heterophilic interaction with αVβ3 integrin [22]. Recent studies have shown that αVβ3 integrin increased in IL-1β-stimulated astrocyte-derived EVs, and blocking of αVβ3 integrin suppressed the uptake of astrocyte-derived EVs into neurons, suggesting the role of αVβ3 integrin in EVs transfer [34,35]. This evidence suggests that DICAM expressed in the exosomal membrane can interact with the αVβ3 integrin of microglia. This interaction between DICAM and αVβ3 integrin can promote the internalization of EVs into microglia, or is directly involved in the activation of αVβ3 integrin signaling. It needs further investigation to confirm the interaction between DICAM and αVβ3 integrin in the EV-mediated microglia regulation.

DICAM-deficient mixed glial cells showed significantly enhanced phosphorylation of p38 MAPK by LPS and IFN-γ stimulation compared to WT, which can be responsible for the proinflammatory phenotypes in the mixed glial cell culture and CRPS model in DICAM KO mice. As this phenotype was not observed in that of the astrocyte culture, the microglia may contribute to this enhanced p38 signaling. Indeed, p38 MAPK signaling was activated in microglia of the spinal cord after peripheral nerve injury, and pharmacologic inhibition of p38 MAPK suppressed tactile allodynia [36,37]. A recent study by Perea et al. revealed that p38 activation in aged mice occurred mainly in microglia, but not in astrocytes and neurons [38]. Moreover, they identified a subpopulation of microglia with low levels of p38 activation, which were rod-shaped and had a less activated phenotype [38]. In an inflammatory milieu, the activation of microglial p38 MAPK is critical for the production of inflammatory cytokines, such as IL-1β and TNFα, through its downstream targets of MAPK-activated protein kinase 2 (MK2) and mitogen- and stress-activated kinase 1 (MSK1) [39]. The inflammatory role of p38 MAPK is also mediated by autophagy suppression in microglia through the inactivation of UNC51-like kinase-1 (ULK1), which is involved in the initiation of the autophagic cascade [40,41]. This raises the following question: how is DICAM-exosome machinery involved in the inhibition of p38 MAPK? Although sufficient data on this topic do not exist, microRNAs such as miR-21 or miR-451 are known to suppress p38 MAPK activation [42,43]. Our findings imply that DICAM-rich EVs can easily transfer these p38-inhibitory miRs into microglia, and consequently promote anti-inflammatory M2-polarization of microglia.

Neuroinflammation characterized by “glial activation” is crucially involved in synaptic plasticity through inflammatory mediators, and consequently in the development of central sensitization [44]. The central glia, especially microglia and astrocytes, have different patterns of activation; microglial activation in the spinal cord is very rapid and dramatic, whereas astrocyte activation is more persistent and occurs in more painful conditions [4,45]. These findings suggest a critical role of microglia in the initiation of central sensitization. Indeed, a study by Ikeda et al. reported that the pharmacologic inhibition of microglia and p38 MAPK with minocycline and SB203580, respectively, attenuated the development of central sensitization in rats subjected to peripheral nerve injury [46]. This result suggests that microglial activity control with DICAM can be an effective strategy for the inhibition of neuroinflammation and subsequent central sensitization.

Our data showed declining trends of the serum DICAM levels in CRPS patients compared to the controls. A previous study investigated the serum level of inflammatory mediators and revealed that inflammatory cytokines, such as IL-8 and sTNFR, and substance P were significantly increased in CRPS type I patients compared to the controls, but adhesion molecules, such as E-selectin, L-selectin, and P-selectin, decreased in CRPS patients [47]. Although it is unknown why the serum levels of adhesion molecules decrease in CRPS patients, DICAM, as an immunoglobulin superfamily cell adhesion molecule, showed a decreasing trend in the serum of CRPS patients. Congruent with our results of the microglia-inhibition effects of DICAM, the decreased serum level of DICAM may be associated with microglia activation in CRPS patients. Further study is needed to confirm whether DICAM levels as a serum marker can predict the severity or clinical course of CRPS.

In conclusion, our findings highlighted that DICAM secreted via astrocyte-derived EVs plays an important role in the M2 polarization of microglia through the suppression of p38 MAPK signaling, and consequent attenuation of neuroinflammation during central sensitization. Further investigations are warranted to evaluate not only its significance as a surrogate marker, but also its potential as a therapeutic target for central sensitization.

## Figures and Tables

**Table 1:** Primer list used in this manuscript.

Gene	Primer	Primer Sequences
Il-1β	Forward	TGA AAT GCC ACC TTT TGA CAG TG
	Reverse	ATG TGC TGC TGC GAG ATT TG
Cxcl10	Forward	CCA AGT GCT GCC GTC ATT TTC
	Reverse	TCC CTA AGG CCC TCA TTC TCA
Il-10	Forward	CTT ACT GAC TGG CAT GAG GAT CA
	Reverse	GCA GCT CTA GGA GCA TGT GG
Cd86	Forward	TCA ATG GGA CTG CAT ATC TGC C
	Reverse	GCC AAA ATA CTA CCA GCT CAC T
Gfap	Forward	AGG CAG AAG CTC CAA GAT GA
	Reverse	TGT GAG GTC TGC AAA CTT GG
Cd68	Forward	TGT CTG ATC TTG CTA GGA CCG
	Reverse	GAG AGT AAC GGC CTT TTT GTG A
Arg	Forward	ACA TCA ACA CTC CCC TGA CAA
	Reverse	TAC GTC TCG CAA GCC AAT GTA
Cd11b	Forward	CTG GTG CTC TTG GCT CTC AT
	Reverse	GGC AGC TTC ATT CAT CAT GT
Gapdh	Forward	AGC CCA AGA TGC CCT TCA GT
	Reverse	CCG TGT TCC TAC CCC CAA TG

**Table 2:** The information of patients with CRPS who were included in this study.

	Age	Sex	Onset Duration	NRS	Pain Site
1	81	Male	32	8	Lt arm
2	55	Male	40	7	Rt arm
3	65	Male	39	9	Lt arm
4	54	Male	32	6	Lt arm
5	48	Female	32	7	Lt leg

NRS; numeric rating scale.

## Data Availability

The datasets used and/or analyzed during the current study are available from the corresponding author upon reasonable request.
